# Non invasive hemodynamic monitoring for fluids and blood resuscitation during placenta praevia accreta cesarean delivery: a retrospective observational study

**DOI:** 10.1186/s44158-022-00083-2

**Published:** 2022-12-27

**Authors:** Maria Loreto, Massimo Pisanti, Marco Celentani, Gilda Pasta, Alfredo Erman, Claudio Santangelo, Luca Gregorio Giaccari, Pasquale Sansone, Romolo Villani

**Affiliations:** 1 Hospital of National Relevance “A. Cardarelli”, Naples, Italy; 2 Department of Economics, Universidad Carlos III de Madrid, Madrid, Spain; 3 National Cancer Institute IRCCS - “G. Pascale” Foundation, Naples, Italy; 4 Fatebenefratelli “S. Giovanni di Dio” Hospital, Naples, Italy; 5 University of Campania “Luigi Vanvitelli”, Naples, Italy

**Keywords:** Hemodynamic monitoring, Goal-directed therapy, Placenta Praevia

## Abstract

**Background:**

We carry out a retrospective observational analysis of clinical records of patients with major placenta praevia who underwent cesarean section surgery over a period of 20 months in our hospital. Out of a total of 40 patients, 20 were subjected to Goal-Directed Therapy (GDT) implemented with non-invasive hemodynamic monitoring using the EV1000 ClearSight system (*Group I*) and 20 to standard hemodynamic monitoring (*Group II*). Given the risk of conspicuous blood loss, this study evaluate the impact on maternal and fetal health of GDT relative to standard hemodynamic monitoring.

**Results:**

Average total infusion of fluids was 1600 +/− 350 ml. Use of blood products occurred in 29 patients (72,5%), of which 11 had a hysterectomy and 8 were treated with Bakri Balloons. For 2 patients > 1000 mL of concentrated red blood cells were used. When stroke volume index SVI dropped below 35 mL/m2/beat, it responded well to the infusion of at least 2 crystalloid boluses (5 ml/kg) in 7 patients. Cardiac index (CI) increased in 8 patients in concomitance with a reduction in medium arterial pressure (MAP), but the use of ephedrine (10 mg iv) re-established acceptable baseline values. Group I means are higher than Group II means for MAP, lower for RBC usage, end-of-surgery maternal lactates and fetal pH, and for LOS. Statistical analysis determines that the null hypotheses of equalities between Groups I and II can be rejected for all measures apart from MAP at baseline and induction. Proportions of serious complications in Groups I and II are respectively 10% and 32% and Boschloo’s test rejects the null of equality of proportions against the alternative hypothesis of lower proportion of occurrence in Group I than in Group II.

**Conclusions:**

Hypovolemia can lead to vasoconstriction and inadequate perfusion with decreased oxygen delivery to organs and peripheral tissues and ultimately cause organ dysfunction. Despite the small sample size due to the rarity of the pathology, our statistical analysis finds evidence in favor of more favorable clinical outcomes for patients who received GDT implemented with non-invasive hemodynamic monitoring infusion relative to patients who received standard hemodynamic monitoring.

## Background

According to the Royal College of Obstetricians and Gynecologists (RCOG) classification, [1] Placenta Praevia (PP) is classified as major if it completely covers the Internal Uterine Orifice (IUO). Placenta Accreta (PA) describes situations in which the placenta is pathologically adherent to the uterus due to a lack of decidual layer, leading to an invasion of the myometrium by the chorionic villi [1]. The PA is classified according to the depth of myometrial invasion. Placenta Increta describes cases in which chorionic villi invade the full thickness of the myometrium; Placenta Percreta describes instead cases in which chorionic villi’s invasion reaches the serosa and may involve adjacent organs such as the bladder. Incidence is challenging to determine because diagnosis depends in part on the prevalence of cesarean sections, but it is currently estimated at 2/1000 of pregnancies [1–3].

In patients suffering from this type of pathology, which makes delivery by cesarean section essential, massive blood losses (i.e., greater than 1000 ml) are not unusual, and patients frequently require intra- and sometimes perioperative blood transfusions. This makes fast, reliable and easy to use hemodynamic monitoring potentially valuable [4].

Improvement in patient outcomes has become a significant concern in surgical settings and the use of Goal-Directed Fluid Therapy (GDT) is gaining acceptance for appropriate perioperative fluid/volume management because it is essential for the success of Early Recovery After Surgery (ERAS) protocols. While rarely used in obstetrician surgery, GDT monitoring may be valuable with abnormal placenta implantation. GDT relies on hemodynamic optimization, as fluid titration, inotropes administration, or blood transfusions, to achieve optimal oxygen delivery to tissues. Perioperative fluid overload may cause acidosis, coagulopathy, tissue, and pulmonary edema, while hypovolemia may lead to hypoperfusion, tissue hypoxia, and oxygen debt. It is well known that Mean Artery Pressure (MAP) and Heart Rate (HR) remain stable even with variations of up to 30% of total blood volume and therefore they cannot provide reliable guidelines for fluid therapy. But fluid GDT based on different parameters may improve perioperative outcomes [4–7].

In early 2018, in our hospital a multi-specialist team of gynecologists, radiologists, neonatologists, and anesthesiologists initiated discussion of the development of a standardized diagnostic and therapeutic pathway. This led to a quality of care improvement initiative evaluating the efficacy of a GDT protocol for eligible patients.

The EV1000 ClearSight system enables fundamental advanced hemodynamic parameters combined with continuous non-invasive blood pressure monitoring thanks to a non-invasive finger cuff. Continuous monitoring of advanced hemodynamic parameters like cardiac index (CI), stroke volume (SV), stroke volume variation (SVV), systemic vascular resistance (SVR), and medium blood pressure (MBP) was performed. The non-invasive hemodynamic monitoring made possible by the EV1000 ClearSight system provides data that allow to proactively make clinical decisions throughout the care process, from moderate to high-risk surgical patients, and can also be used in the perioperative phase to manage clinical situations of patients in constant evolution [8–12].

## Aims

The main objective of our study is to assess the benefits of the implementation of a perioperative emo-component utilization and the utility of Goal-Directed Therapy in terms of length of hospital stay, end-of-surgery lactate values and fetal pH, and postoperative complications.

This article contains new scientific contributions compared to any previously published articles on a similar topic as this is the first time that the benefit of non-invasive hemodynamic monitoring in pregnant women with obstetric pathology has been analyzed.

## Materials and methods

A retrospective observational study was carried out collecting and examining the medical records of patients affected by abnormal placental implantation treated between April 2019 and December 2020 in the Obstetrics and Gynecology Unit of the Hospital “A. Cardarelli” (Naples, Italy).

This study was conducted following the STROBE (Strengthening the Reporting of Observational Studies in Epidemiology) guidelines for reporting observational studies [13].

This study was approved by the local ethics committee (approval number 174 – February 2, 2022) and it was in accordance with the Declaration of Helsinki [14]. An informed consent was signed for each patient.

### Inclusion criteria

1. Abnormal placental implantation with signs of placental accretism major;
2. Planned cesarean section procedure;
3. Age of patients between 28 and 42 years old;
4. American Society of Anesthesiology (ASA) Class I or II;
5. Hemoglobin > 11 g/dl.

### Exclusion criteria

1. Pathologies or comorbidities that could interfere with the coagulation structure.

A total of 40 medical records were examined. At the time of cesarean section surgery, patients were subjected to either standard hemodynamic monitoring or to GDT implemented with non-invasive hemodynamic monitoring using the EV1000 ClearSight system. Out of the total of 40 patients, 20 had been subjected to additional monitoring with the EV1000 ClearSight system. This occurred when the anesthesiologist on duty had adequate experience with the system and when the appropriate size (Small, Medium or Large) of the disposable sensor with dedicated cuff suitable was available.

Depending on the hemodynamic monitoring system employed during the procedure we label patients as follows:

- **Group I**: *Patients subjected to standard hemodynamic monitoring together with non-invasive hemodynamic monitoring with the EV1000 ClearSight system;*
- **Group II**: *Patients subjected to standard hemodynamic monitoring.*

### Procedure

**Fig. 1:**
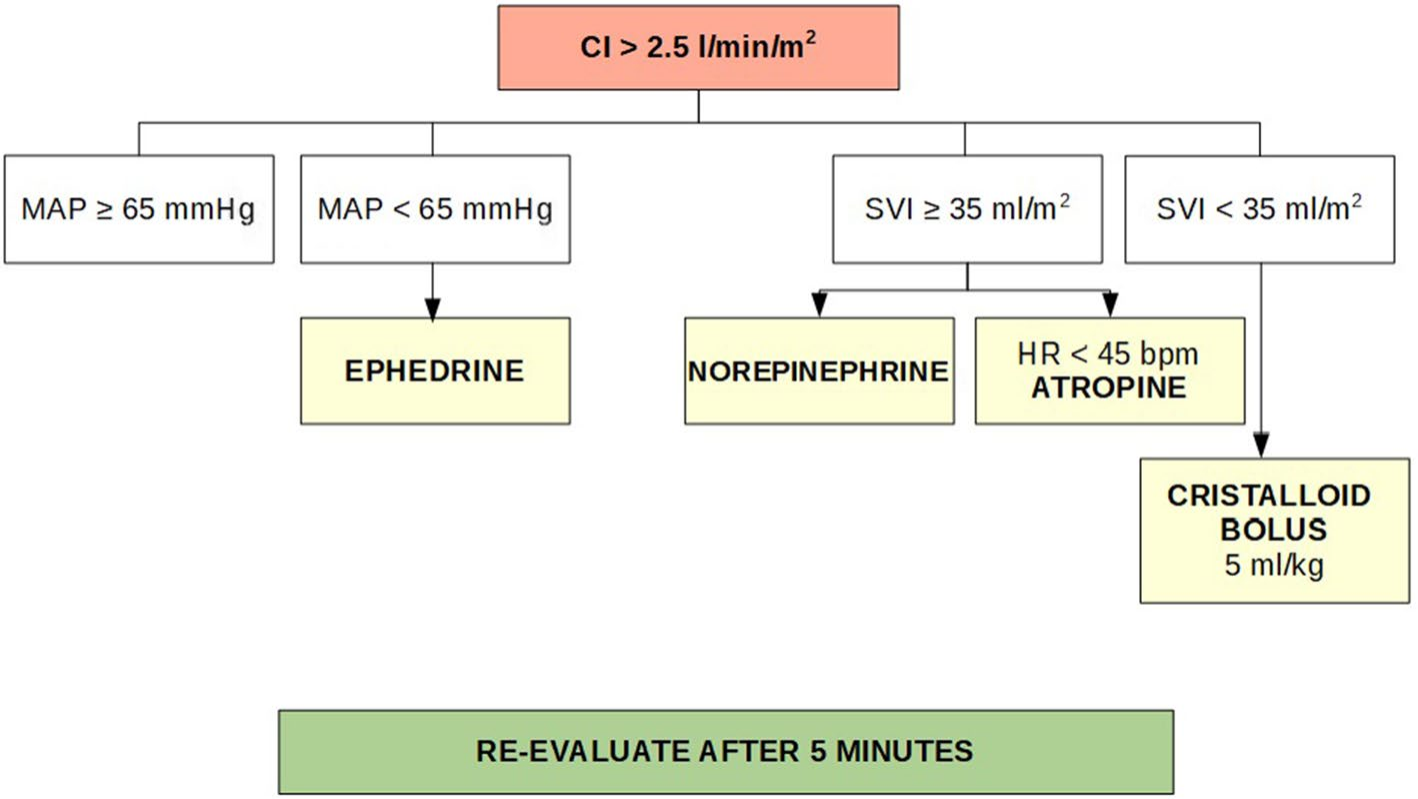
Proactive protocol

The protocol for procedure and data collection that was followed is outlined below:

- Entry of the patient into the operating room.
- Cannulation of 2 large-caliber peripheral vessels.
- Monitoring of basic vital parameters: consciousness, continuous ECG (electrocardiographic trace in DII), HR (heart rate), oximetry, respiratory rate, temperature, diuresis, and metabolic structure: blood gas analysis (BGA) with the determination of maternal lactate at least at the end of the surgery, fetal pH, intraoperative fluids, transfusion of blood components.
- Hemodynamic monitoring. Either of the following:
  - Non-invasive hemodynamic monitoring by EV1000 ClearSight system of:
    - Direct parameters: cardiac index (CI), mean arterial pressure (MAP), stroke volume index (SVI) (or stroke volume variation (SVV) in patients submitted to general anesthesia);
    - Indirect parameters: O2 demand (DO2) and RSVI.
    - Figure 1 describes the proactive protocol that was followed to correct alterations that occurred during surgery.
  - Standard hemodynamic monitoring: continuous ECG, heart rate and systolic/diastolic blood pressure. In this group, hypotension was corrected with fluid bolus therapy until blood pressure normalization.
- Anesthesia:
  - Continuous peridural anesthesia (CPE): For patients with no known contraindications.
  - Blended anesthesia (CPE followed by general anesthesia): For patients when the duration of surgery resulted in poor compliance to CPE alone.
  - General anesthesia: For patients with known absolute or relative contra-indications to loco-regional anesthesia and for some other patients.
- Data collection:
  - Hemodynamic data measurements (CI, SVI, MAP, DO2) and lactates were logged at baseline (**T0**), induction (**T1**), post-extraction (**T2**), intermediate (**T3**), and end-of-surgery (**T4**).
  - Body temperatures, diuresis, fluid and blood products administration, fetal pH, complications (such as persistent fever, delayed channeling, mild to severe respiratory failure, wound infection or dehiscence, thrombotic event, etc.), length of hospital stay (LOS) and 30-day hospital readmission.

### Statistical analysis

Statistical analysis was conducted using different statistical tests based on the need to determine the meaning of a value obtained in the whole population or to compare data obtained between treated and untreated patients (Software used R version 4.0.3–2020-10-10; Packages: dplyr, car, one waytests, exact). The outcomes showing departures from normal values were evaluated by Shapiro-Wilk normality test, F-test, Levene’s test, two-sample t-test, Welch two-sample t-test, and Wilcoxon sum test.

The t-test was used to determine the means of two sets of data significantly different from each other. The independent samples t-test is used when two separate sets of independent and identically distributed samples are obtained, one from each of the two populations being compared.

## Results

The clinical characteristics of the patients in the two groups are similar: all ASA class I and II patients with placenta previa accreta.

Descriptive statistics for all 40 patients are reported in Table 1. This table reportsm the clinical data recorded during the intervention: the number of bags of concentrated red blood cells used, the fetal pH at birth, the maternal lactates at the end of the intervention, the days of hospitalization. In addition, some events were recorded during the operation or in the postoperative period indicated in the table with appropriate abbreviations: B: bakri balloon, C: coagulation disorder, D: delayed channeling, E: embolization F: fever H: histerectomy M: mild respiratory failure S: severe respiratory failure V: vascular access thrombosis W: wound dehiscence. Of these events, only those that caused complications in the postoperative period were considered and indicated with a score of 1.

**Table 1:** Descriptive Statistics

Patient		Group	MAP T0	MAP T1	MAP T2	MAP T3	MAP T4	Red blood cells -unit	Length of hospital stay	Fetal pH T4	Maternal lactates T4	Serious perioperative complications	Recorded Events
1		I	63	61	53	44	55	2	7	7.31	1.40	0	B
2		I	84	72	74	84	90	2	5	7.28	0.40	0	H
3		I	76	71	64	54	75	3	6	7.32	0.70	0	BF
4		I	84	73	72	85	68	2	5	7.32	0.20	0	
5		I	64	60	68	71	80	3	7	7.28	1.00	1	HC
6		I	74	72	52	58	60	2	6	7.26	0.80	0	
7		I	90	77	68	63	63	1	5	7.32	0.90	0	F
8		I	75	55	69	60	65	2	4	7.29	0.90	0	B
9		I	80	76	85	80	71	0	3	7.26	0.80	0	
10		I	84	80	76	74	69	0	6	7.32	0.50	0	BH
11		I	69	52	69	65	65	1	3	7.28	0.60	0	
12		I	75	45	40	66	65	2	6	7.24	1.40	0	F
13		I	70	60	56	64	68	4	10	7.32	1.60	1	BHV
14		I	84	70	78	77	81	1	4	7.40	1.00	0	
15		I	89	77	76	62	65	0	5	7.34	1.50	0	F
16		I	88	85	84	86	84	0	3	7.39	1.20	0	
17		I	88	85	80	87	85	0	4	7.36	0.80	0	
18		I	77	90	71	60	62	0	5	7.32	1.60	0	
19		I	86	72	87	77	81	0	4	7.28	0.60	0	
20		I	85	64	54	66	78	0	7	7.31	1.20	0	EFH
	**Average Group I**		79.25	69.85	68.80	69.15	71.50	1.25	5.25	7.31	0.96	10.0%	
	**StDev Group I**		8.27	11.69	12.46	11.81	9.61	1.25	1.71	0.04	0.41		
21		II	54	50	36	44	48	3	6	7.33	1.30	0	H
22		II	70	66	62	64	65	2	10	7.30	0.90	1	W
23		II	72	66	50	48	52	4	9	7.26	1.30	0	BH
24		II	68	62	60	64	66	1	4	7.31	0.60	0	
25		II	66	60	56	54	58	2	5	7.25	0.80	0	
26		II	90	78	70	68	66	2	7	7.28	1.70	1	DW
27		II	72	64	48	46	52	4	8	7.31	1.50	1	CH
28		II	82	76	68	66	65	0	3	7.34	1.10	0	
29		II	78	65	58	56	55	2	7	7.27	1.30	1	S
30		II	88	78	60	58	66	3	6	7.35	1.40	0	W
31		II	86	78	64	44	58	5	7	7.32	1.80	1	BCH
32		II	78	65	60	64	68	1	4	7.34	0.80	0	
33		II	85	76	66	64	66	0	3	7.33	1.10	0	
34		II	68	58	55	58	65	3	5	7.29	1.30	0	H
35		II	80	70	68	65	60	1	4	7.24	1.50	0	
36		II	91	82	54	58	60	2	6	7.34	1.40	1	M
37		II	66	58	55	60	63	2	5	7.28	0.80	0	
38		II	64	60	55	65	65	0	3	7.30	1.10	0	
39		II	72	66	40	46	55	6	9	7.29	1.60	1	BHS
40		II	84	60	69	74	83	0	5	7.34	1.20	0	
	**Average Group II**		75.70	66.90	57.70	58.30	61.80	2.15	5.80	7.30	1.23	31.6%	
	**StDev Group II**		10.02	8.59	9.21	8.78	7.65	1.69	2.09	0.03	0.33		

Legend for Perioperative complications: *B* Bakri balloon; *C* Coagulation disorder; *D* Delayed channeling, *E* Embolization; *F* Fever; *H* Hysterectomy; *M* Mild respiratory failure; *S* Severe respiratory failure; *V* Vascular access thrombosis; *W* Wound dehiscence

The patients treated with continuous epidural anesthesia were 32. Only one patient received general anesthesia (being in treatment with high doses of heparin and being operated in an emergency regime). Seven patients received blended anesthesia (continuous epidural anesthesia and subsequent general anesthesia) when the duration of surgery resulted in poor compliance to continued epidural anesthesia alone. Average duration of procedures was 115 ± 35 minutes.

The MAP remained > 50 mmHg in most patients, in fact only two patients in group I and two patients in group 2 had a MAP < 50 mmHg after placental extraction. Total infusion of fluids was on average 1600 ± 350 ml.

For 3 patients (7.5%), bleeding was quickly controlled after the extraction of the placenta, and no containment maneuvers were necessary. For 8 patients (20%) placing a Bakri Balloon was necessary to control uterine bleeding. For 11 patients (27.5%) hysterectomy was practiced to stop bleeding. Finally, for 7 patients (17.5%), an interventional radiologist was required for selective embolization of uterine arteries. In all patients, blood losses were greater than 1500 ml.

Use of blood products occurred in 28 patients (72.5%): 12 in Group I and 16 in Group II. Ten patients received more than 2 units of blood units (25%), 3 in Group I (15%) and 7 in Group II (35%). Only 2 patients (5%) received > 1000 mL of concentrated red blood cells. Hysterectomy was necessary for a total of 11 patients, of which 5 in Group I (25%) and 6 in Group II (30%).

Fetal pH was 7.31 ± 0.04 in Group I and 7.30 ± 0.03 in Group II. At the end of surgery patients had 0.96 ± 0.41 lactates, while maternal lactates were 1.23 ± 0.33 in Group II.

72.5% of patients were discharged within 7 days. Postoperative length of hospital stay was between 7 and 9 days for 11 patients (4 in Group I versus 7 in Group II): 3 patients with thrombocytopenia or other coagulation factors defects, 1 patient with thrombotic complication after femoral artery cannulation, and 7 patients who required intensive postoperative care. Serious perioperative complications occurred 9 times, of which 7 in Group II.

**Fig. 2:**
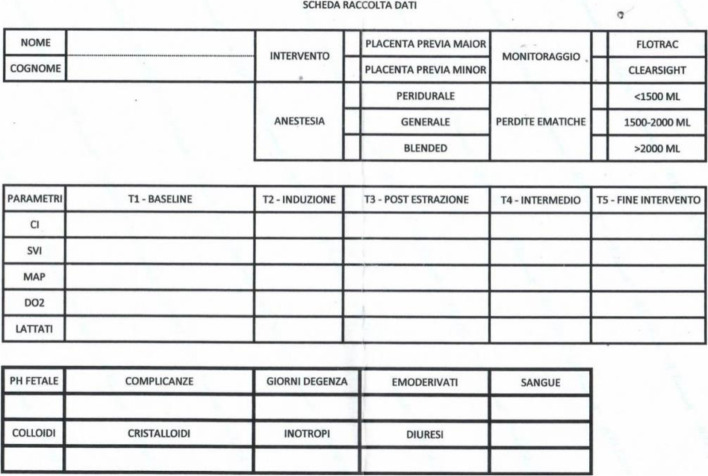
Group I Patients Cardiac Indices (CI), T0-T4

**Fig. 3:**
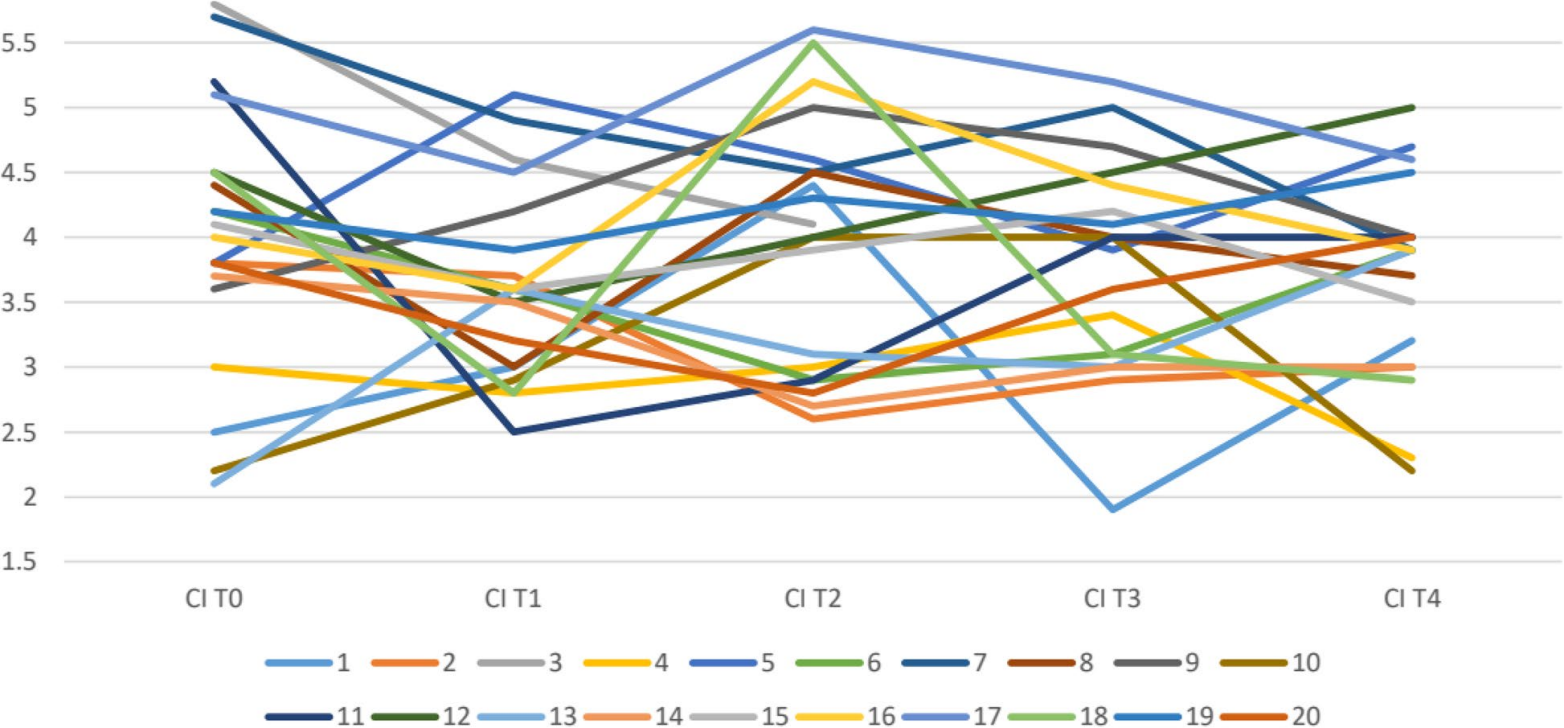
Group I Patients Stroke Volume Indices (SVI), T0-T4

**Fig. 4:**
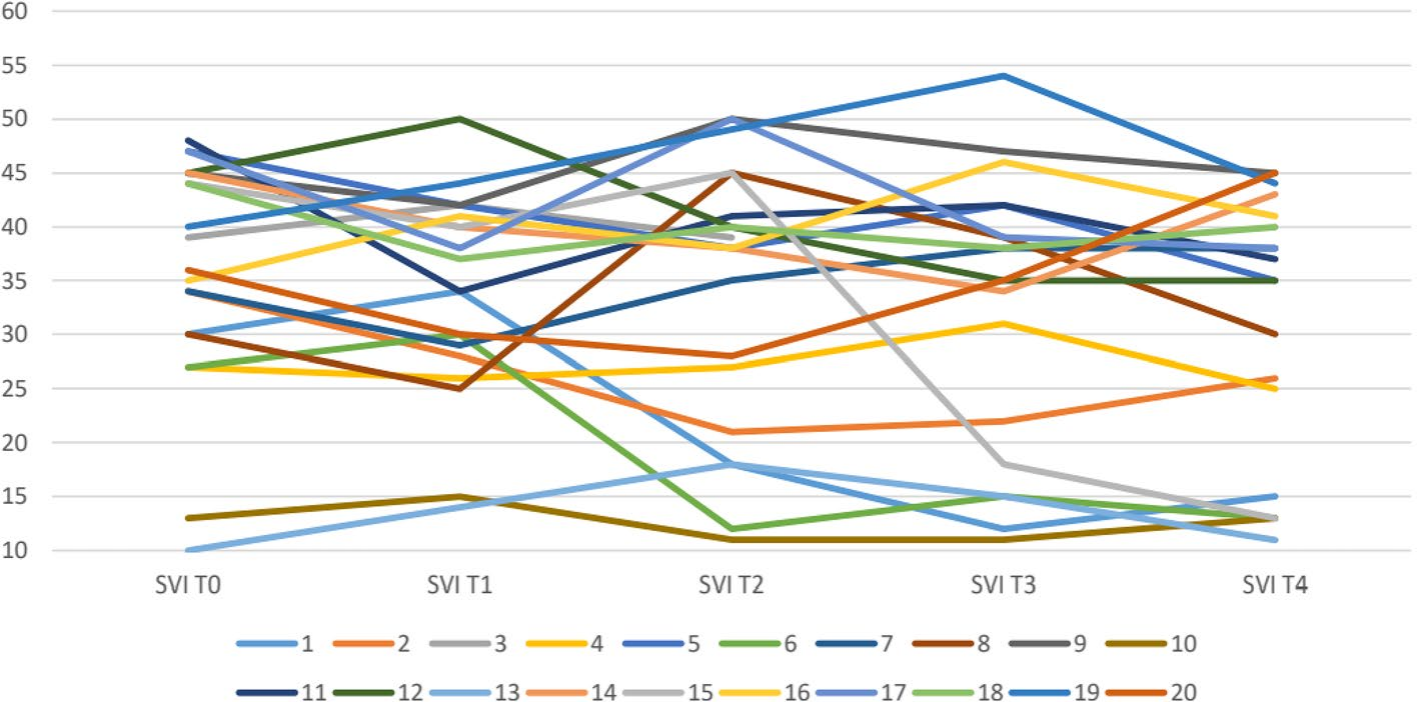
Group I Patients Oxygen Delivery (O2D), T0-T4

The clearsight hemodynamic monitoring data recorded in the patients of Group I were collected in Tables 2, 3, 4 and represented in Figs. 2, 3, 4. The hemodynamic values were promptly corrected by recording alterations of the parameters for short periods of time with consequent maintenance of an adequate perfusion during all phases of the surgery, even the most critical.

**Table 2:** Group I Patient Cardiac Index

Patient	CI T0	CI T1	CI T2	CI T3	CI T4
1	2.5	3	4.4	1.9	3.2
2	3.8	3.7	2.6	2.9	3
3	5.8	4.6	4.1		4
4	3	2.8	3	3.4	2.3
5	3.8	5.1	4.6	3.9	4.7
6	4.2	3.6	2.9	3.1	3.9
7	5.7	4.9	4.5	5	3.9
8	4.4	3	4.5	4	3.7
9	3.6	4.2	5	4.7	4
10	2.2	2.9	4	4	2.2
11	5.2	2.5	2.9	4	4
12	4.5	3.5	4	4.5	5
13	2.1	3.6	3.1	3	3.9
14	3.7	3.5	2.7	3	3
15	4.1	3.6	3.9	4.2	3.5
16	4	3.6	5.2	4.4	3.9
17	5.1	4.5	5.6	5.2	4.6
18	4.5	2.8	5.5	3.1	2.9
19	4.2	3.9	4.3	4.1	4.5
20	3.8	3.2	2.8	3.6	4

**Table 3:** Group I Patient Stroke Volume Indices (SVI) T0-T4

Patient	SVI T0	SVI T1	SVI T2	SVI T3	SVI T4
1	30	34	18^a^	12^a^	15^a^
2	34	28	21	22	26
3	39	42	39		53
4	27	26	27	31	25
5	47	42	38	42	35
6	27	30	12^a^	15^a^	13^a^
7	34	29	35	38	38
8	30	25	45	39	30
9	45	42	50	47	45
10	13	15	11	11	13
11	48	34	41	42	37
12	45	50	40	35	35
13	10	14	18	15	11
14	45	40	38	34	43
15	44	40	45	18^a^	13^a^
16	35	41	38	46	41
17	47	38	50	39	38
18	44	37	40	38	40
19	40	44	49	54	44
20	36	30	28	35	45

^a^ SVV after patient was submitted to general anesthesia

**Table 4:** Group I Patient Oxygen Delivery (DO2) T0-T4

Patient	O2D T0	O2D T1	O2D T2	O2D T3	O2D T4
1	968	668	673		753
2	1071	647	873	875	633
3	888	780	750	830	824
4	1032	980	948	888	868
5	925	856	789		896
6	890	832	620	698	743
7	1012	817	856	804	844
8	1141	768	588	704	789
9	874	690	665	724	784
10	753	642	584	698	700
11	1012	990	738		878
12	744	678	380		612
13	633	784	866		799
14	926	658	726		786
15	847	706	688	680	716
16	889	679	698	700	786
17	848	882	840		884
18	916	890	767	999	1189
19	966	930	888	843	
20	860	800	670	720	880

The SVI dropped below 35 mL/m2/beat in 7 patients (35%), but it responded well to the infusion of at least two crystalloid boluses (5 ml/kg); and the CI in 8 patients (40%) increased with a reduction in MBP, but the use of ephedrine (10 mg iv) re-established acceptable baseline levels.

All recorded data were subjected to statistical analysis to assess their significance. The statistical analysis of the results (through two simple t-tests at the usual confidence intervals) gave significant results as regards the maintenance of adequate blood pressure values in the times T3 (*p*-value 0.002), T4 (p-value 0.002) and T5 (p-value 0.001) and also of the lactate value at the end of surgery (p-value 0.025).

One side test where the null is rejected at the usual confidence level (0.05) for blood unit (*p*-value = 0.0318). Through Boschloo’s test, the data examined the association of two Bernoulli distributed random variables, which is a uniformly more powerful alternative to Fisher’s exact test for postoperative complications. Indeed true differences in proportions of occurrence of treated versus untreated are less than 0 for postoperative complications (p-value 0.04) and consequently of the length of hospital stay (p-value 0.026) in the group of patients undergoing non-invasive monitoring with the EV1000 ClearSight system.

## Discussion

The comparison of outcome markers showed a statistically significant reduction between the two groups. The clinical outcome was favorable in patients who received an infusion of goal-directed fluids and were subjected to a proactive protocol with non-invasive hemodynamic monitoring EV1000 ClearSight system. Hypovolemia can lead to vasoconstriction and inadequate perfusion, with decreased oxygen delivery to organs and peripheral tissues, causing organ dysfunction. On the other hand, fluid overload can lead to interstitial edema, local inflammation and likely impair the regeneration of collagen, thus negatively affecting tissue healing while increasing the risk of wound dehiscence, wound infections, and anastomotic leakage. Therefore, maintenance of intravascular good volume throughout the perioperative period is ideal. The principle behind GDT is to maximize tissue oxygen delivery without fluid overload by achieving measurable optimal hemodynamic indices. Surprisingly, the total quantity of infused liquids was not significantly different between the two groups, as it is strongly influenced by the experience of the anesthetist present for this type of intervention.

The difference in the amount of red blood cells used is statistically significant. Periods of intraoperative hypotension were reduced, as was the use of blood products which occurred in less than 50% of patients.

The comparison of outcome markers such as maternal lactates to end-surgery and perioperative complications showed a statistically significant reduction between the two groups.

Only a minimum number of patients had an extension of hospitalization times, and none of the patients had complications or 30-day readmission, splanchnic, hepatological, and renal microcirculation failure.

Hemodynamic stability guarantees adequate tissue generation and a metabolic balance to reduce postoperative complications, whereas the prolongation of the days of hospitalization due to insufficient microcirculation consequents the onset of suture infections, fever, and renal insufficiency [15–23]. The presence of statistically significant data regarding the results not affected by the method makes this study interesting. However, only its application in a more substantial number of patients can provide direct results in choosing the most appropriate monitoring.

Perioperative fluid management have received increased interest in recent years [24]. The validity of ClearSight System may depend on the clinical situation such as the type of surgery or the patient’s clinical condition. Several studies have investigated the effectiveness of non-invasive hemodynamic monitoring in various fields, such as vascular surgery [25], cardiac surgery [26], bariatric surgery [27] and neuroendovascular interventions [28]. All concluded that non-invasive hemodynamic monitoring is

a good option for hemodynamic monitoring during induction of anesthesia. The ClearSight system provided accurate measurements of arterial blood pressure compared with invasive methods. In accordance to these studies, it is a safe and reliable alternative for invasive blood pressure monitoring during different surgical procedures. On the other hand, it is not reliable in critically ill patients [29] and patients undergoing neurosurgery in a sitting position [30].

### Limitations

The study has a series of limitations related to the small sample in which it was conducted. However, the obstetric pathology of the abnormal placental implant represents a low percentage of obstetric patients. We managed to reach this number of patients by being the reference hospital for the treatment that can be performed by a multidisciplinary team of specialists with various therapeutic options (for example, embolization of the uterine branches with the interventional radiologist, assistance to the premature newborn with the Neonatological Intensive Care, dedicated anesthetist, etc.). Another limitation was represented by the fact that the study was not conducted in a blind matter. However, we only selected patients with similar inclusion criteria precisely to avoid being influenced by the type of monitoring to be used, and the use of the monitoring provided for a one-to-one random selection. The statistical analysis various tests were used in order to have a more reliable confirmation of the data obtained.

## Conclusions

Perioperative hemodynamic management based on the evaluation of advanced hemodynamic variables aims at optimizing cardiovascular dynamics to improve the patient’s postoperative outcome.

In our study we had encouraging results in terms of intraoperative hemodynamic management and maternal-fetal outcome. Due to the small study size, further research is needed to evaluate and establish non-invasive hemodynamic monitoring for hemodynamic management during placenta praevia accreta cesarean delivery.

## Data Availability

The datasets used and analyzed during the current study are available from the corresponding author on reasonable request.

## Abbreviations

ASA: American Society of Anesthesiology
BGA: Blood gas analysis
CI: Cardiac index
CPE: Continuous peridural anesthesia
DO2: O2 demand
ERAS: Early Recovery After Surgery
GDT: Goal-Directed Fluid Therapy
HR: Heart Rate
LOS: Length of hospital stay
MAP: Mean Artery Pressure
MBP: Medium blood pressure
PA: Placenta Accreta
PP: Placenta Praevia
RCOG: Royal College of Obstetricians and Gynecologists
SV: Stroke volume
SVR: Systemic vascular resistance
SVV: Stroke volume variation
